# Research status and hotspots on the mechanisms of liver X receptor in cancer progression: A bibliometric analysis

**DOI:** 10.1097/MD.0000000000037126

**Published:** 2024-03-29

**Authors:** Yukun Chen, Siqi Deng, Jiexia Xu, Yu Yan, Shuwen Lan, Mingzhang Guo

**Affiliations:** a Fujian University of Traditional Chinese Medicine, Fuzhou, China; b College of Traditional Chinese Medicine, Fujian University of Traditional Chinese Medicine, Fuzhou, China.

**Keywords:** bibliometric analysis, cancer, CiteSpace, liver X receptor, visual analysis, Vosviewer

## Abstract

**Background:**

The mechanism of liver X receptor in cancer has been gradually revealed in recent years. This study is committed to analyzing the current research status of the mechanism of liver × receptor in cancer progression by using bibliometric methods and to explore the development trend of liver × receptor related research in the future, in order to provide some reference for further exploration in this field.

**Methods:**

The Web of Science core collection database was used to carry out the original data retrieval. Excel software was used for data statistics. Vosviewer and CiteSpace software were used to analyze the publication situation, cooperation network, reference co-citation, keyword and term co-occurrence, term bursts, and cluster analysis, and draw visual maps.

**Results:**

A total of 631 publications meeting the research criteria were included by December 2022, with an average of 32.5 citations per paper. The main research fields were molecular biology, oncology and cell biology, and the papers were mainly published in journals about molecular, biology and immunology. Cell is the journal with the highest citation. The United States is the most influential country, the University of California, Los Angeles is the main research institution, and Gustafsson, Jan-ake is the author with the highest output. In reference co-citation clustering, cluster#2 “cancer development” is the main cluster, and the period from 2014 to 2018 is an important stage of relevant theoretical progress. “Tumor microenvironment” with high burst and novelty became the most noteworthy term in term burst.

**Conclusion:**

Using bibliometric methods to reveal the current status of LXR and cancer mechanisms, and making predictions of possible future hotspots based on the analysis of the current situation, the translation of LXR anti-cancer research to clinical applications, the impact on the tumor microenvironment as a whole and more immune pathways, and the formation of a systematic cognition of the effects of more cancer cell lines and oncogenic signaling crosstalk, which is a possible direction for future research.

## 1. Introduction

For a long time, cancer has been an important factor in global morbidity and mortality. Especially in recent years, with the rising trend of global aging, the global incidence and mortality of cancer have increased rapidly. In the future, the global medical burden brought by cancer will still increase.^[1]^ Although with the improvement of medical level and the popularization of medical screening, the survival time, quality of life, and prognosis of patients with malignant tumors have been improved to some extent, the treatment of various cancers is still a difficult problem in clinical practice. Active research on the mechanism of cancer progression and new methods of treatment to enhance the efficacy and improve the quality of survival of patients are major challenges that modern medicine and related disciplines need to face together.

In recent years, it has been continuously found that lipid metabolism is involved in the process of tumor growth, proliferation, invasion and migration, and the reprogramming of lipid metabolism becomes an important marker of malignancy.^[2,3]^ Increased lipogenesis in the tumor microenvironment and the storage and mobilization of intracellular lipid droplets are important components that constitute cancer-related lipid changes^[4]^ and show a strong correlation in cancer progression.

The liver X receptor (LXR) was first identified as acting in a mechanism involved in the transport and elimination of cholesterol.^[5]^ As the relationship between lipid metabolism and cancer progression has been revealed, the maintenance of active signaling in cancer cells is dependent on high-intensity endogenous cholesterol biosynthesis and lipid particle uptake. This metabolic mechanism is tightly regulated by a cholesterol homeostatic network that includes the LXR, low-density lipoprotein particle receptor (LDLR) and sterol response element binding protein (SREBP),^[6]^ the mechanism of LXR in cancer cell cholesterol metabolism is expected to be a new target for many types of cancer. There is also an increasing number of studies showing that LXR is also involved in regulating multiple signaling pathways^[7–9]^ as well as participating in tumor immunity^[10,11]^ and other ways involved in tumor progression. Therefore, it is of great significance to systematically review the relevant research and key areas of LXR in cancer progression and discuss the possible future research trends.

Bibliometrics is an interdisciplinary science that uses mathematics and statistics to comprehensively evaluate and analyze certain knowledge carriers in order to summarize the research status of a certain field. The use of bibliometric methods in the evaluation of medical papers and the construction of quantitative evaluation systems through objective indicators is a widely used scientific research method^[12]^ that helps to quickly and objectively establish a scientific understanding of a subject area and to visually analyze its development. This research aims to establish an understanding of the current status and hotspots of the research on the mechanism of LXR and cancer by analyzing the bibliometric method, and to make a judgment on the possible future development trend, with a view to making a useful exploration of new strategies for cancer research.

## 2. Materials and methods

### 2.1. Data source

The original data of this study were extracted from the Web of Science Core Collection (WoSCC), one of the most commonly used databases for bibliometric analysis studies at present, which can provide information on authors, journals, affiliations, countries/regions, annual publication amount, research areas of papers.

### 2.2. Search strategy

In order to retrieve and collect the literature information needed for the study more accurately, the retrieval strategy used in this study was an all-field search. Retrieval formula: (ALL = (LXR OR “Liver X receptor” OR “Liver X-activated receptor” OR “Hepatic X receptor” OR Nr1h3 OR Nr1h2) AND ALL = (Cancer OR Neoplasms OR Carcinoma OR Tumor OR Tumor malignant OR malignancies)). A total of 1173 publications were retrieved by the strategy. A total of 1173 articles were retrieved, and those that did not fit the purpose of the study were excluded by the researchers. The search and screening work was carried out independently by 2 researchers, and the results were cross-checked, with a third researcher joining in to make a judgment on the disputed part of the results.

### 2.3. Screening conditions

Publication time: Based on the publication time of WoSCC, documents published since the establishment of the library until December 2022 are included. Type: publications classified as Articles are included, and other types of publications are excluded. Language: publications in English are included and non-English publications are excluded. Content: Exclude literature whose titles and abstracts are not relevant to the topic of this study, or literature whose content is not related to LXR and cancer.

### 2.4. Data collection

The original data were exported from selected publications on the filtered WoSCC database in the form of “plain text file - full record and cited references” as the data source of CiteSpace6.1.6, and in the form of “TAB delimited file - full record and cited references” as the data source of Vosviewer.

### 2.5. Analytical method

CiteSpace software (Chaomei Chen, Drexel University, USA) has the functions of identifying co-cited authors and references, capturing high burst keywords, generating time zone views, and visual presentation of co-occurrence results. Used CiteSpace6.1.6 to import data sources, create a research project after removing duplicates, set the time slice as 1 year, the pruning settings were selected as “pathfinder” and “pruning sliced networks,” then ran the project for analysis.

Vosviewer (the Centre for Science and Technology Studies, Leiden University) generates an intuitive visual network from the co-citation relationships of the literature and the co-occurrence of keywords and generates a visual data map by parameter adjustment. This study also used Pajek software to assist in adjusting the node network generated by Vosviewer for better clustering presentation and node placement.

Some of the relevant data statistics generated by the software will be exported to Excel for further organization and presentation in graphical form.

In the generated node network, each node represents an item, and the size of the node is positively correlated with the strength of the item (e.g., number, citation frequency, etc.); the link between different nodes indicates the existence of a correlation between nodes, and the thickness of the connection line indicates the strength of the association; closely related nodes are often arranged closer together to facilitate the generation of cluster views. A bright purple box around a node indicates that the centrality of the node is greater than 0.1 and is marked as a key node, suggesting that the node may be the central hub node associated with multiple clusters or types.

## 3. Results and discussion

**Figure 1.**
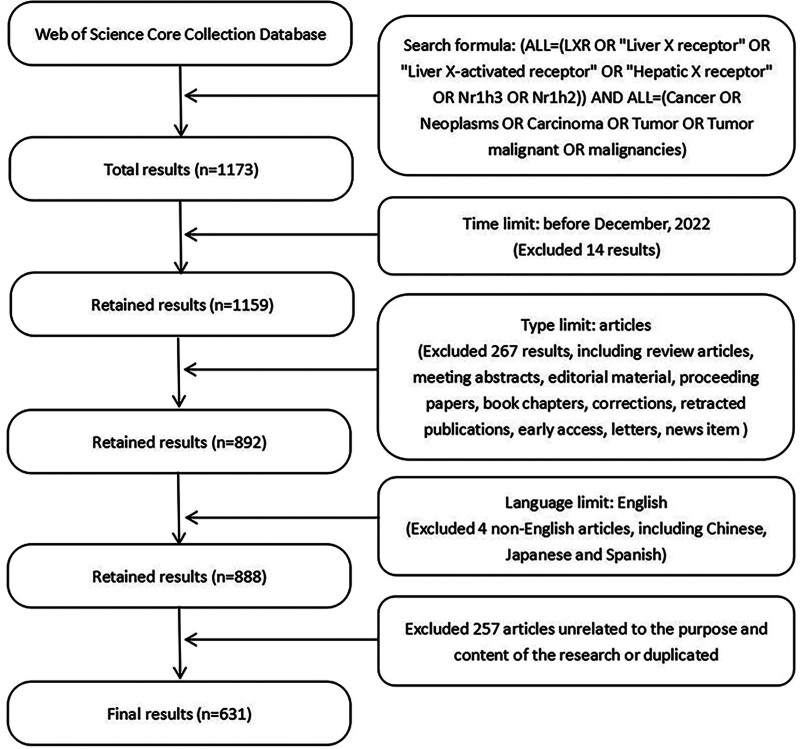
The flow chart of literature screening.

A total of 1173 articles were retrieved in this field by using the search formula, and we finally obtained 631 eligible articles according to the filtering criteria to exclude the results that did not meet the requirements, as shown in Figure 1.

### 3.1. Analysis of annual publications and journals

**Figure 2.**
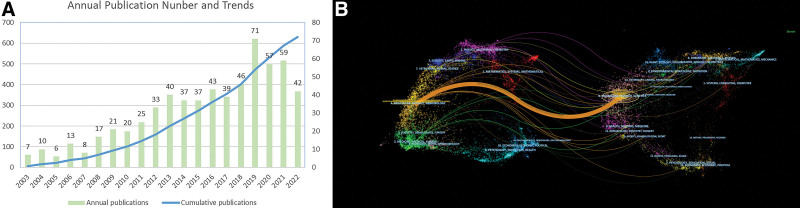
(A) The annual number of publications and the cumulative number of publications in this field; (B) A dual-map overlays visualization of the journal created based on CiteSpace software.

The results of the analysis of the WoSCC database using Excel software (Fig. 2A) showed that the annual number of articles in the field related to liver X receptors and cancer increased from 7 articles in 2003 to 71 articles in 2019, and although the number of articles decreased from 2019 to 2022, it still maintained a high number level, and the cumulative number of articles showed a more stable growth trend in general. The 631 articles retrieved were cited 20,537 times in total, and the average article was cited 32.5 times. The relatively stable growth trend represents the development space and research heat of this research field.

In terms of research areas, the top 3 research areas in the literature were biochemistry molecular biology, oncology, and cell biology, which occupied 24.41%, 20.60%, and 16.16% of the research, respectively, and the rest of the top 10 research areas in terms of number are shown in Table 1.

**Table 1:** The top 10 research areas in terms of number.

Rank	Research areas	Publication counts	Percentage	Centrality	Degree
1	Biochemistry Molecular Biology	154	24.41	0.54	16
2	Oncology	130	20.60	0.34	12
3	Cell Biology	102	16.16	0.26	13
4	Endocrinology Metabolism	59	9.35	0.07	6
5	Pharmacology Pharmacy	56	8.87	0.29	10
6	Multidisciplinary Sciences	53	8.40	0	1
7	Medicine Research Experimental	48	7.61	0.19	11
8	Immunology	41	6.50	0.29	13
9	Gastroenterology Hepatology	27	4.28	0	2
10	Biophysics	26	4.12	0.03	3

From the source of published journals, the publications included in this research were from 297 journals. and the top 5 journals in terms of number of publications and citations (Tables 2 and 3) had the highest number of publications for PLOS ONE (n = 19) and the highest number of citations for CELL (n = 1730). The dual-map overlays visualization of journals was generated based on the CiteSpace software (Fig. 2B), in which the labels represent the research fields included in the journals, the left part is the journals publishing in the research field, the right part is the journals included in the references in the journals, and the curves from left to right are the citation lines. As can be seen from the figure, most of the papers included in this research were published in molecular, biology, immunology and other journals, mainly citing molecular, biology, and genetics journals.

**Table 2:** The top 5 of journals in terms of publications.

Rank	Source	Counts	Total citations	Average citation per item	Total link strength	IF (2022)	Quartile in category
1	Plos One	19	487	25.6	65	3.7	Q2
2	Biochemical and Biophysical Research Communications	14	275	19.6	69	3.1	Q3
3	Scientific Reports	13	195	15	51	4.6	Q2
4	Journal of Biological chemistry	12	973	81.1	59	4.8	Q2
5	International Journal of Molecular Sciences	11	154	14	15	5.6	Q1

**Table 3:** The top 5 of journals in terms of citations.

Rank	Source	Counts	Total citations	Average citation per item	Total link strength	IF (2022)	Quartile in category
1	Cell	5	1730	346	152	64.5	Q1
2	Journal of Biological Chemistry	12	973	81.1	59	4.8	Q2
3	Gastroenterology	4	705	176.3	52	29.4	Q1
4	Oncogene	5	538	107.6	81	8	Q1
5	Proceedings of the National Academy of Sciences of the United States of America	9	516	57.3	49	11.1	Q1

The research on the mechanism of LXR and cancer is concentrated in the field of molecular biology and oncology, which is also evidenced by the types of journals published. In addition to comprehensive journals containing a wide range of genres, other journals with high impact or high publication counts show more advantages in the fields of molecular biology and oncology. For example, Cell, as a top journal covering the field of experimental biology such as molecular biology and oncology, is the main journal found to be high-influence in the present study. In Figure 2B, we found that this study also has a certain amount of publications in the field of medicine and clinical medicine, and partially intersects with the field of molecular biology and oncology. No matter what discipline type of cancer research, it is ultimately to return to the purpose of medical and human health services, so our team believes that such cross-referencing can reflect the application transformation level of scientific research and clinical medicine to a certain extent, although the intensity of such cross-referencing curve is insignificant compared with the self-induction in the fields of molecular biology and oncology. This just shows that the existing experimental research still has a lot of room for development in the process of clinical transformation, which may become one of the research directions worth promoting in the future.

### 3.2. Countries and institutions situation analysis

**Figure 3.**
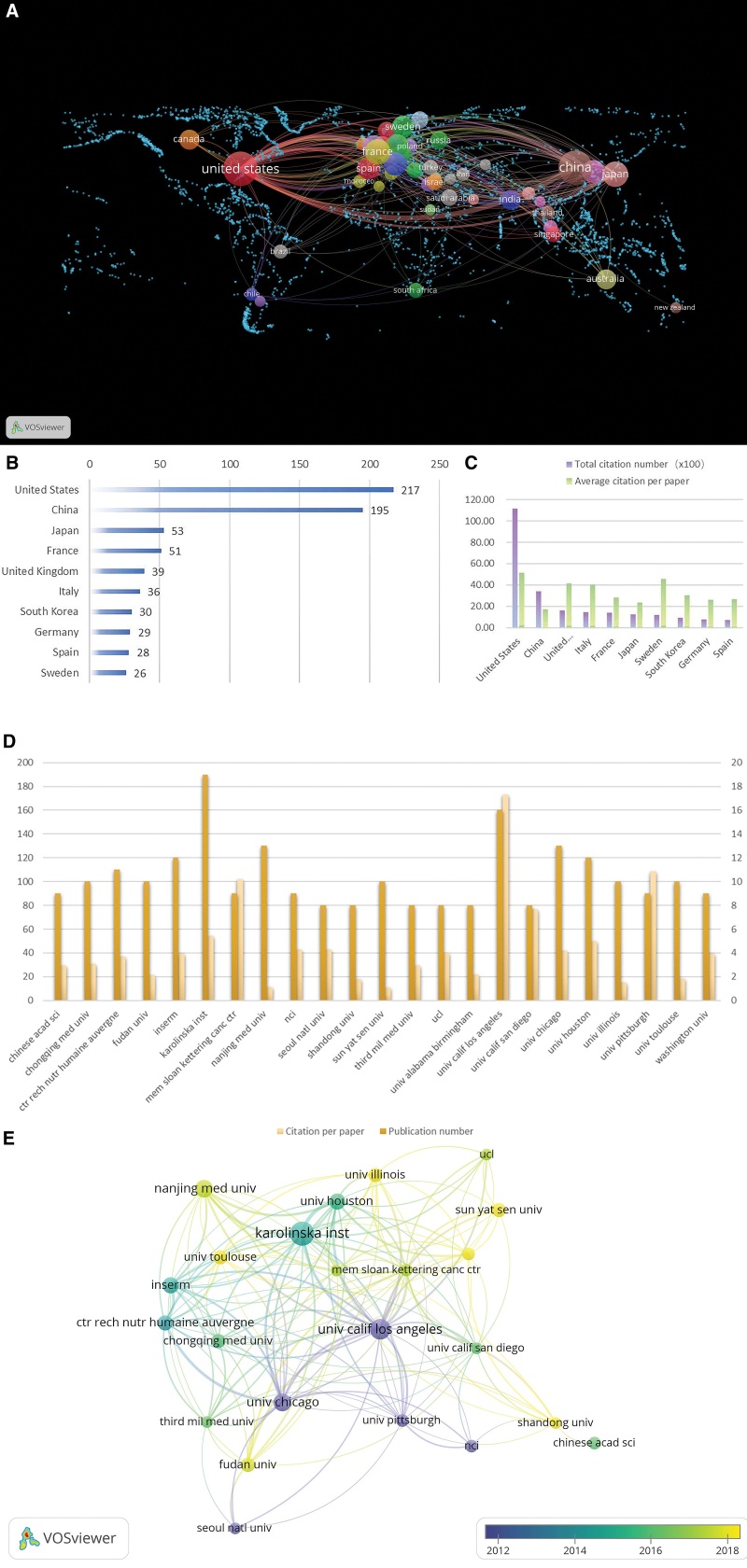
(A) The global geographic view of countries and regions participating created by Vosvierer; (B) Statistics on the number of publications of participating countries; (C) A statistical chart of the number of total citation and average citation; (D) The statistics of the number of publications and citations of the institutions; and (E) A time view of the network of reference relationships between institutions.

A global geographic view of countries and regions participating in the study created based on Vosviewer (Fig. 3A) shows that 50 countries have been involved in the publication of relevant studies, some of which were done by researchers from several different countries. The country with the highest number of publications was the United States (n = 217), followed by China (including China and Taiwan, n = 195), Japan (n = 53), France (n = 51), the United Kingdom (n = 39), Italy (n = 36), South Korea (n = 30), Germany (n = 29), Spain (n = 28), and Sweden (n = 26) (Fig. 3B).

In terms of total citations (Fig. 3C), the country with the highest number of total citations is the United States (n = 11,155), which far exceeds the other countries, followed by China (n = 3419), which also reaches more than twice the number of other countries. As to the average number of citations, the United States (n = 51.41), Sweden (n = 45.77), the United Kingdom (n = 41.54), and Italy (n = 40.72) possess a higher average number of citations. The US has the highest number of publications, citations, and citations per article, far ahead of other countries, which reflects the dominant position of the US in this research field. In addition, although China has a huge number of publications and citations after the US, it has the lowest average number of citations among the top 10 countries, which indicates that China’s high citations are based on a high number of publications, but the overall quality of articles still needs to be improved.

The institution analysis is independent of the country-region analysis, which can objectively grasp the strength of the institution in the relevant research progress. A total of 1038 institutions are involved in research related to the mechanisms of LXR and cancer. Institutions with at least 8 publications and at least 100 citations are defined as active institutions in the relevant field, with a total of 23 institutions making significant contributions in the field (Fig. 3D). Karolinska Institute (n = 19), University of California, Los Angeles (n = 16), University of Chicago (n = 13), and Nanjing Medical University (n = 13) had more published papers, while the University of California, Los Angeles, University of Pittsburgh, Mem Sloan Kettering Cancer Center had higher average citations. It indicates literature from these institutions has a higher authority. Using Vosviewer to analyze the citation relationships among these institutions and plotting the citation network time view (Fig. 3E) shows that the average time of publication of literature at institutions such as UCLA, the University of Pittsburgh, and the University of Chicago is mainly concentrated in the early years, and that these institutions provide an important foundation for the study of LXR-related mechanisms.

From the institutional analysis, many of the high-impact institutions in this field were published in the early years, suggesting that there may have been a wave of research related to this field in the early years and established a more influential theoretical foundation. It is worth noting that a significant proportion of the more recent studies with some influence are from China, suggesting that the focus of Chinese research in this field may be on the latest cutting-edge research and promoting the development of the field, which may also partially explain the low average citation in China. We tried to sort out relevant clues from the time node in Figure 3E. The high number of published papers and citations of UCLA was our entry point. According to the mark of 2012 as the time range, we found that a considerable degree of highly cited papers came from this institution during the period from 2008 to 2010. These papers^[13,14]^ provide a solid foundation for LXR as an important regulatory hub for cholesterol metabolism and successfully correlate LXR signaling with immune responses through LXR-dependent sterol metabolism, suggesting that LXR acts primarily through lipid metabolism and modulation of immune pathways. This period of research on the role and properties of LXR set the tone for subsequent scientific progress and provided the theoretical basis for the proposal of LXR as a therapeutic target for tumors, and scholars from the Karolinska Institute and the University of Houston were the key promoters of this new concept.^[15]^ These early important research results were mainly driven by institutions from the United States, while in the subsequent period, many institutions from China, such as Chongqing Medical University, Nanjing Medical University, Sun Yat-sen University, Fudan University, etc., have conducted many studies based on this foundation, and these projects^[16–19]^ focus more on the impact of LXR as an activation target on multiple types of cancer and multiple pathways. According to the time view and our search results, the period was positioned around 2014-2018, which is the most booming period of research on LXR and cancer, with multiple types of cancers being revealed to be affected by the regulation of LXR activation, and China was able to become the second country after the United States in terms of the number of papers published.

### 3.3. Author citation analysis

**Figure 4.**
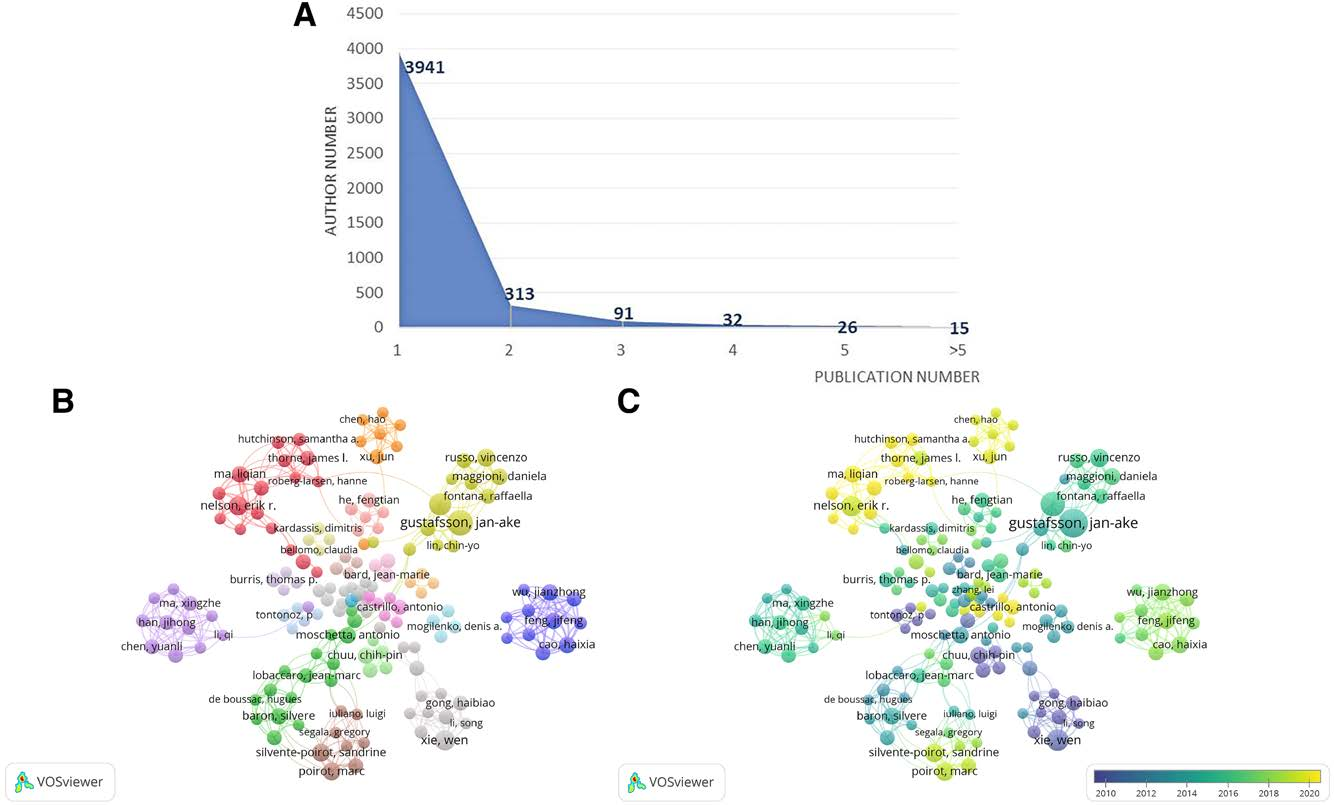
(A) The statistics of the number of authors and the number of publications; (B and C) The author citation network and time view respectively.

A total of 4418 authors participated in the research on the mechanism of LXR and cancer, of which 89.2% published only 1 article (Fig. 4A). Among the top fifteen authors with the number of publications and citations (Tables 4 and 5), Gustafsson, Jan-ake (n = 14) published the most articles, followed by Steffensen, Knut r. (n = 11), Xie, Wen (n = 9), Nelson, Erik r. (n = 8), and the most cited author was Tontonoz, P (n = 1222), followed by Xie, Wen (n = 977), Pei, Lm (n = 961), Lee, Jung hoon (n = 797). The author collaboration network created based on Vosviewer (Fig. 4B and C) shows that some of the more active or high-impact authors tend to have close collaborative ties and form stable research teams, such as the team of Xie, Wen, Lee, Jung hoon and Wada, Taira, the team of Tontonoz, P, Pei, Lm, Castrillo, A, Joseph, Sb, the partnership between Gustafsson, Jan-ake and Steffensen, Knut r., the partnership between Nelson, Erik r. and Ma, Liqian.

**Table 4:** The top 15 authors with the number of publications.

Rank	Author	Publication counts	Total citations	Average citation per item	H-Index	Total link strength
1	gustafsson, jan-ake	14	758	54.14	10	79
2	steffensen, knut r.	11	609	55.36	10	121
3	xie, wen	9	977	108.56	8	60
4	nelson, erik r.	8	640	80	6	76
5	poirot, marc	7	118	16.86	5	49
6	russo, vincenzo	7	313	44.71	5	53
7	silvente-poirot, sandrine	7	118	16.86	6	49
8	baron, silvere	6	164	27.33	6	32
9	cao, haixia	6	62	10.33	6	53
10	chuu, chih-pin	6	322	53.67	6	9
11	feng, jifeng	6	50	8.33	6	46
12	ma, liqian	6	65	10.83	4	73
13	maggioni, daniela	6	288	48	6	52
14	moschetta, antonio	6	460	76.67	6	39
15	wu, jianzhong	6	62	10.33	6	53

**Table 5:** The top 15 authors with the number of citations.

Rank	Author	Publication counts	Total citations	Average citation per item	H-Index	Total link strength
1	tontonoz, p	4	1222	305.5	4	10
2	xie, wen	9	977	108.56	8	60
3	pei, lm	3	961	320.33	3	7
4	lee, jung hoon	5	797	159.4	4	29
5	umetani, michihisa	3	793	264.33	3	13
6	wada, taira	4	789	197.25	3	27
7	gustafsson, jan-ake	14	758	54.14	10	79
8	castrillo, a	3	748	249.33	3	9
9	joseph, sb	3	748	249.33	3	9
10	parks, john s.	3	696	232	3	1
11	edwards, peter a.	2	650	325	2	17
12	nelson, erik r.	8	640	80	6	76
13	he, jinhan	3	635	211.67	3	18
14	steffensen, knut r.	11	609	55.36	10	121
15	park, sunghee	3	608	202.67	3	16

In the author citation analysis, several stable collaborative teams or close collaborative relationships were formed among high-impact or high-activity scholars. Among them, Xie, Wen’s team and Tontonoz’s team had an important influence on early studies. Xie, Wen et al focused on the mechanism of the role of nuclear receptors including LXR and liver fatty acid transporter protein CD36 in lipid homeostasis^[20,21]^ and early studies on prostate cancer,^[22]^ which may serve as a precursor to studies related to lipid homeostasis in cancer progression. The studies of Tontonoz et al mainly focused on the regulation of the mechanism of action between LXR and macrophages,^[10,23]^ which may become an important basis for the research of LXR and tumor immune response. The research by Gustafsson, Jan-ake et al involved LXR-mediated processes of cell metabolism, proliferation, immune response and inflammatory response^[15,24]^ and revealed the mechanism by which its regulation of lipid and cholesterol metabolism affects the proliferation of breast cancer cell lines.^[25]^ The studies by Nelson, Erik r et al are on average newly published and focus on the mechanism of action of LXR and its ligand (endogenous cholesterol derivative) 27-hydroxycholesterol (27-HC) in selected cancers such as ovarian and breast cancer^[26,27]^ and reveal the relationship between cholesterol homeostasis and the pathological progression of these cancers. The high-quality research results formed by these stable groups can also be summarized into 2 major directions: cholesterol metabolism and tumor immunity. Combined with the viewpoints of the analysis of nations and institutions, we believe that these are the 2 dominant directions in the current research status of LXR and cancer mechanisms, and there are still many contents waiting to be explored in the future.

### 3.4. Reference analysis

**Figure 5.**
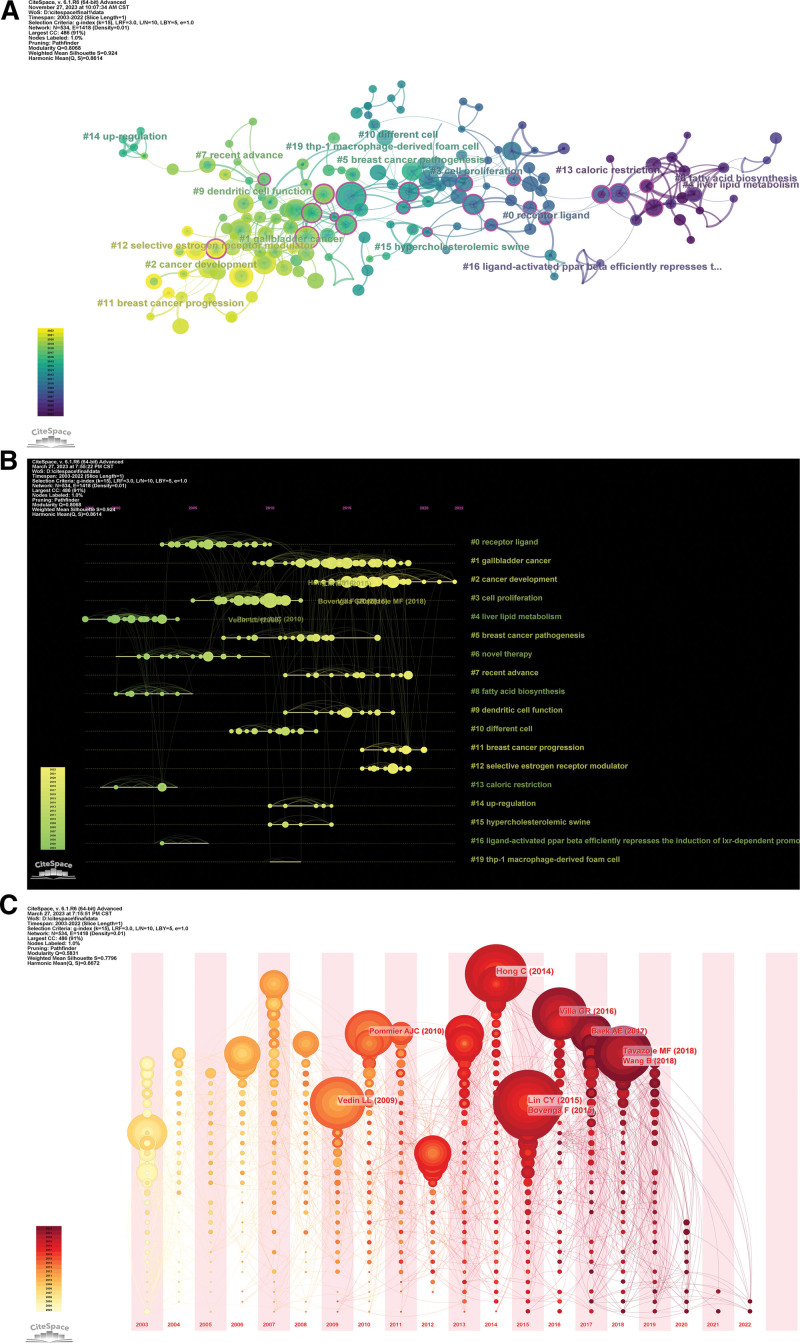
(A) The reference co-citation analysis clustering map; (B) The clustering timeline view of reference co-citation analysis; (C) The time zone view of reference nodes.

Co-citation references are those cited by more than 2 cited references in the literature. The co-citation of some references in different research fields may suggest that these references serve as the common theoretical basis for the derived research directions. Therefore, the co-citation analysis of references can reveal the research basis of this field to a certain extent. The clustering view of the reference co-citation network generated from the reference co-citation analysis based on CiteSpace software (Fig. 5A and B) shows that there are 18 clusters in the main co-citation map, with cluster #1 gallbladder cancer, cluster #2 cancer development, and cluster #3 cell proliferation being the main clusters, and cluster #2 cancer development is the newest cluster. According to the reference co-citation time zone view (Fig. 5C), 2014 to 2018 is a high-frequency period for reference co-citation, with a large size and number of nodes, which indicates that this period is an important development stage for the theoretical basis of LXR and cancer research. This is consistent with the conclusions reached in the analysis of nations and institutions.

In the clustering of references, cluster #1 is labeled as gallbladder cancer in both LLR and LSI, and the key citation in the cluster^[28]^ focuses on a review of the mechanisms of cholesterol homeostasis and cholesterol efflux mechanisms in cancer, based on the high affinity of cancer cells for cholesterol for the purpose of regulating cancer progression. LXR is an important signal in the cholesterol homeostasis system, and studies based on this type of article reveal that LXR affects cancer mechanisms by influencing cholesterol homeostasis and promoting cholesterol efflux. In addition, the highly cited literature of this cluster^[29]^ also basically revolves around the review of the role of LXR in lipid and cholesterol homeostasis. Cluster #2 was labeled by LLR as an emerging player and by LSI as cancer development, and this cluster may contain important new studies on the mechanisms of LXR and cancer. Its key literature^[30]^ indicates that the cholesterol metabolite 27-HC acts as an LXR ligand for cholesterol efflux through the LXR pathway to maintain cholesterol homeostasis, and based on this mechanism may also cause associated signaling and inflammatory responses, which in turn affect cancer. In addition, the highly cited literature in this cluster^[31]^ also involves the study of LXR regulation of innate immunosuppression for antitumor effects.

### 3.5. Keyword and term analysis

#### 1.3.5.. 6A.

**Figure 6.**
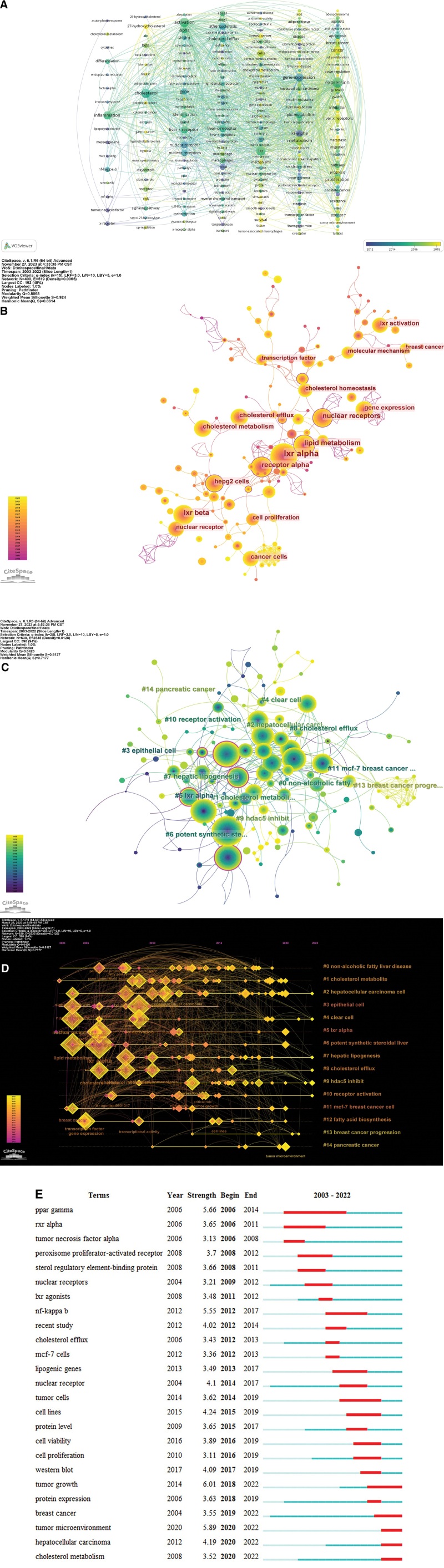
(A) The Keywords co-occurrence map in Y-axis arrangement with the time view; (B) The term co-occurrence view; (C) The term co-occurrence cluster view; (D) The clustering timeline view of term co-occurrence based on (C); (E) 25 terms with strongest occurrence bursts based on CiteSpace.

Keywords are the central words that highly summarize the content of an article. Through the co-occurrence analysis of keywords in different papers, the statistics and analysis of the research areas of the articles can be carried out more accurately and conveniently. Vosviewer was used to analyze the co-occurrence of keywords in 631 papers, and a keyword co-occurrence map was generated (Fig. 6A). Each vertical column of Figure 6A represents a cluster. The words with the highest frequency were “expression” (n = 157), “lxr” (n = 113), “activation” (n = 112), “cholesterol” (n = 110), and “metabolism” (n = 98). The words with centrality greater than 0.1 were “gene expression” (0.19), “lxr alpha” (0.17), “expression” (0.13), “nuclear receptor” (0.1), and “apoptosis” (0.1). The keyword co-occurrence time view generated according to the average time (Fig. 6A) showed that some keywords related to cancer, tumor microenvironment, and fatty liver appeared late, which suggested the novelty of related research and may reflect the research hotspots in recent years.

Terms are specialized words extracted from the title, abstract, references, and other information of an article. Compared with keywords, terms can more comprehensively summarize the research information contained in an article. Based on the term extraction function of Citespace, we made a term co-occurrence analysis on the included literature (Fig. 6B), among which “lxr alpha” was the term with the highest frequency (Table 6), followed by “lipid metabolism,” “lxr beta,” “nuclear receptors.” The centrality of the 5 words “hepg2 cells,” “lxr beta,” “receptor alpha,” “abca1 expression,” “nuclear receptor,” “lipid metabolism” and “gene expression”are greater than 0.1 (Table 7). The clustering view of term co-occurrence (Fig. 6C and D) shows that the term network can be grouped into 15 clusters, of which clusters #0 nonalcoholic fatty liver disease, #1 cholesterol metabolites, #2 hepatocellular carcinoma cell, #7 hepatic lipogenesis, #8 cholesterol efflux, and #9 HDAC5 inhibit are among the important clusters and have gained a certain popularity in recent years.

**Table 6:** Top 20 terms with highest frequency.

Rank	Term	Year	Frequency	Degree	Rank	Term	Year	Frequency	Degree
1	lxr alpha	2006	143	21	11	hepg2 cells	2008	34	47
2	lipid metabolism	2004	58	33	12	nuclear receptor	2004	33	31
3	lxr beta	2009	58	43	13	cell proliferation	2010	33	21
4	nuclear receptors	2004	52	28	14	cholesterol homeostasis	2010	33	33
5	receptor alpha	2006	50	45	15	breast cancer	2004	29	22
6	lxr activation	2009	43	18	16	lxr agonists	2008	29	36
7	cholesterol efflux	2006	42	29	17	transcription factor	2005	29	22
8	cholesterol metabolism	2008	39	34	18	molecular mechanism	2009	29	14
9	gene expression	2005	37	41	19	protein expression	2006	26	37
10	cancer cells	2011	35	32	20	signaling pathway	2004	23	17

**Table 7:** 7 terms with centrality greater than 0.1.

Rank	Term	Year	Centrality	Degree	Frequency
1	hepg2 cells	2008	0.16	47	34
2	lxr beta	2009	0.12	43	58
3	receptor alpha	2006	0.12	45	50
4	abca1 expression	2006	0.11	29	15
5	nuclear receptor	2004	0.10	31	33
6	lipid metabolism	2004	0.10	41	58
7	gene expression	2005	0.10	33	37

Burst detection is used to detect the increase in the frequency of bursts of nodes in a certain period, which suggests that the node represents a research hotspot and frontier in that period. 25 high burst results were obtained using the term burst detection as shown in Figure 6E. The blue part represents the period when the term appeared and the red part represents the duration of the burst. “tumor growth” (n = 6.01), “tumor microenvironment” (n = 5.89) possess high burst intensity. Early burst terms include “ppar gamma,” “rxr alpha,” “tumor necrosis factor alpha,” and in recent years, more cutting-edge burst terms include “tumor growth,” “protein expression,” “breast cancer,” “tumor microenvironment,” “hepatocellular carcinoma,” “cholesterol metabolism.” It is worth noting that “tumor microenvironment” is a term that has only emerged in recent years and has shown high explosive intensity since its emergence, reflecting its importance as a frontier research hotspot.

In the analysis of keywords and terms, we found that the high-frequency word “expression” appeared frequently in all the tests of keywords and terms, and the related derivatives included gene expression, various types of protein expression, etc., which indicated that various types of physicochemical effects under the microscopic point of view were the core of the research on LXR and cancer, and it also conformed to the positioning of the research field; and the high-frequency words “cholesterol” and “metabolism” were in line with the recognition of LXR in the research. It is worth noting that the term “activation” points to the study of LXR receptor agonists, which have the ability to specifically activate the LXR receptor and up-regulate the expression of LXR in order to explore its effect on a certain pathologic mechanism, and the agonistic ligands include endogenous cholesterol derivatives, such as 27-HC, and synthetic products, such as T0901317, GW3965, etc. Relevant nodes can be found in the co-occurrence network. The study of receptor agonists is a breakthrough in the transformation of LXR anti-cancer strategy from theory to clinical application. The emergence of relatively new terms such as “tumor prognosis” and “survival” in the node network indicates that LXR anti-cancer has received attention in clinical treatment, but obviously, there is still more room for development. Exploring the mechanisms and effects of multiple cancer types based on the activation of LXR is an important idea for research in this field, and it is also the core argument that LXR can be used as a targeted protein for cancer treatment, which will be enumerated in the Advances and Frontiers in Cancer Research. As for the latest process in the research, keywords such as breast cancer, tumor supportive therapy, cancer prognosis, hepatocellular carcinoma, tumor microenvironment, tumor-associated macrophages, and autophagy appeared relatively recently in terms of the average occurrence time of word frequency, and the results of term analysis and terms burst also indicate the frontier of relevant content. Keywords and terms such as lipid or cholesterol metabolism are found throughout the period. Therefore, we believe that the relevant studies on the mechanism of LXR and cancer progression are based on lipid metabolism and cholesterol homeostasis, and further derived studies on the mechanism of LXR in various cancers and its role in tumor immunosuppression, which is the current research status in this field.

LXR is a member of the nuclear receptor superfamily, including 2 subtypes LXRα (NR1H3) and LXRβ (NR1H2). The former is mainly expressed specifically in the human liver, intestine, kidney, and adipose tissue, while the latter is commonly expressed in multiple human tissues and organs. LXR can be activated by endogenous cholesterol derivatives or synthetic activators as ligands or plays a regulatory role through the formation of heterodimers between its subtypes and retinol X receptors.^[32]^ LXR was first found to function as a systemic cholesterol sensor, Early research evidence showed that^[33]^ LXRα knockout mice had cholesterol lipid deposition that was difficult to metabolize on a high-cholesterol diet, and exhibited an abnormal rise in low-density lipoprotein (LDL)/high-density lipoprotein. Subsequent studies confirmed that^[13,34]^ activation of LXR mobilizes systemic cholesterol efflux and effectively regulates cholesterol metabolism in the body through the mechanism of LDL receptor (LDLR) expression that reduces cholesterol uptake and promotes its conversion to bile acids, and early studies focused on this aspect. On this basis, studies on LXR and diseases related to high cholesterol such as atherosclerosis, hypercholesterolemia, obesity, and nonalcoholic fatty liver disease were derived, which became part of the important studies in the medium term. During the screening of the literature for this study, our researchers screened out many relevant studies in these areas, especially in the section on atherosclerosis. And with the revelation of the role of lipids and cholesterol in cancer progression, it has also gradually given rise to studies on the possible role of LXR in cancer progression, typified early, especially by prostate cancer, in which LXR has been shown^[35,36]^ to play an important regulatory role, and LXR is also considered by many scholars to be a potential target for many types of cancer in the future.

### 3.6. Cholesterol homeostasis and cancer

Cholesterol, a class of lipid molecules, is an important component of the cell membrane. In addition, cholesterol is responsible for transport and signaling inside and outside the cell membrane, maintaining the integrity of basic cell membrane functions. However, excessive intracellular cholesterol levels can cause some cytotoxicity, so it is important to maintain moderate intra- and extracellular cholesterol levels to maintain certain homeostasis. The mechanism of intracellular cholesterol signal transduction may also play a role in cancer proliferation, and studies have shown that in^[37]^ cancer progression is often accompanied by an imbalance in cholesterol homeostasis, and the rapid proliferation of cancer cells with high intensity of active signaling during cancer progression is dependent on the predatory demand for endogenous cholesterol synthesis and lipid uptake. SREBP2 regulates the transcriptional process of the enzyme encoding cholesterol synthase to promote the de novo synthesis of cholesterol, while high levels of cholesterol in turn inhibit the action of SREBP2 forming a negative feedback regulation of cholesterol homeostasis.^[38]^ In contrast, LXR forms a positive feedback regulation of metabolic cholesterol by regulating the transfer of intracellular cholesterol into high-density lipoprotein through the encoded ATP-binding cassette transport proteins ABCG1 and ABCA1. LXR agonists are also involved in cholesterol metabolism through ABCG5 and ABCG8 stimulating the cholesterol biliary excretion pathway. In addition, LDLR, a major mediator of cholesterol uptake, is similarly regulated by the SREBP2 and LXR pathways, and LXR reduces LDLR levels by increasing the inducible degrader of LDLR.^[39]^ These reactions together constitute the regulatory network of cholesterol homeostasis. Cholesterol is synthesized mainly through the mevalonate pathway, starting with the combination of acetyl coenzyme A. Intermediate products such as mevalonate, farnesyl pyrophosphate, and geranyl geranyl pyrophosphate appear in the process. These products play important roles in oncogenic signaling pathways such as PI3K/Akt/mTOR and Wnt/β-catenin pathways,^[40–42]^ and thus cancer tissues often show an urgent need for de novo synthesis of cholesterol during cancer progression, and LXR controls this progression by converting acetyl coenzyme A-based cholesterol synthesis into fatty acid synthesis. Cholesterol is also involved in the formation of lipid rafts, an important structure on the surface of cancer cell membranes that hosts many relevant proteins involved in cancer promotion and regulation of apoptosis, and LXR-regulated cholesterol inhibition can affect the formation of lipid rafts,^[38]^ which in turn inhibits cancer cell function.

### 3.7. LXR and tumor immunity

Tumor microenvironment (TME) refers to the non-cancerous components of cancer, including various factors released by cancer cells. The tumor microenvironment interacts with tumor tissue cells, while mediating crosstalk and interactions between non-cancerous and cancerous tissues, and is an important factor in the development of tumor cells. In the emergence of terms, tumor microenvironment shows a very representative high popularity and research frontier, and the emergence of relatively new terms such as tumor-associated macrophages is expected to become the focus of new research. Immune cells are the main components of the tumor microenvironment, which interact with tumors and influence a series of processes such as tumor growth, proliferation and metastasis. Macrophages in TME, called tumor-associated macrophages (TAM), are among the most common cells in infiltrating TME, and they usually exhibit M2-like polarization. Such polarization shows support for tumor growth, including promoting tumor angiogenesis, inhibiting tumor immune response to enhance tumor escape, and promoting tumor metastasis. Is associated with a poor prognosis of cancer.^[43]^ The immunosuppression of TAM is reflected in the inhibitory downregulation of T cell proliferation activation, NK cell activation, and CD8 + T cell lysis activity to construct an immunosuppressive environment for infiltrating TME. The current studies show^[44,45]^ that LXR negatively affects various immunosuppressive mechanisms of TAM, including promoting the repolarization tendency of macrophages, down-regulating chemokine CCL7 expression to down-regulate regulatory T cells, inhibiting TAM precursor program and interfering with TAM suppression T cell process thus disrupting its immunosuppressive environment establishment. Natural killer T cells (NKT), a subgroup of T lymphocytes, also possess some surface markers of NK cells, which enable them to recognize tumor-associated lipid antigens and participate in anti-tumor immunity, and related studies have shown^[46]^ that both subtypes of LXR are involved in the immune response of iNKT and participate in the growth and development of NKT cells through the PLZF signaling pathway or other independent signals. Multiple innate and adaptive immune mechanisms in TME are also restricted by myeloid-derived suppressor cells (MDSC) leading to poor outcomes under tumor immunotherapy, whereas activation of LXR can target apolipoprotein E (ApoE) to significantly down-regulate the survival rate and number of MDSC, while driving cytotoxic T cell immune responses,^[33]^ which also reflects another anti-tumor potentiating mechanism of LXR by lifting the restriction on innate immunity. However, it is controversial that^[25]^ the oxysterol ligand activation pathway of LXR inhibits CCR7 gene expression on mature dendritic cells, limiting their migration to lymphoid organs via the LXR-dependent pathway and thus producing an anti-tumor immune suppressive effect, and tumor cells may therefore produce the relevant ligands leading to immune escape. It is also reasonable to speculate whether LXR receptor agonist activation exerts an anticancer mechanism that also partially antagonizes the oxysterol activation pathway, and further studies on this controversy are expected to be the next phase of research. Both the prominent lipid metabolism mechanisms and the immune mechanisms of LXR have been revealed in cancer-related studies, but it is important to note that the antitumor effects of the immune properties of LXR are not isolated from the lipid metabolism effects. Tumor cells interact with normal cells in the TME pathway, and this crosstalk is often accomplished in a lipid-mediated manner. The growth and proliferation of adaptive immune response T cells depend on the regulation of sterol content by LXR, and the anti-tumor immune effects of NKT cells depend on the recognition of lipid antigens.^[47]^ High levels of tumor cell lipid uptake activate the PI3K pathway and thus induce M2-like polarization of TAM.^[48]^ The potent effect of MDSC on immunosuppression is also derived from the phosphorylation response generated by the reprogramming of lipid metabolism and tumor lipid accumulation.^[49]^ This suggests that cancer progression is a network containing multiple complex mechanisms interconnected with each other, and the role of LXR in cancer progression deserves a more systematic view.

### 3.8. Advances and frontiers in cancer research

Through the visualization map of this study, we found that a wide range of cancers are involved in LXR regulation, encompassing prostate, breast, hepatocellular, ovarian, colon, pancreatic, and melanoma cancers. All of these disease names can be found in the node network of keywords or terms, especially breast cancer and hepatocellular carcinoma, and in the clustering of terms and the detection of the emergence of key terms, we found that there are separate important clusters and more cutting-edge outbreaks for both breast cancer and hepatocellular carcinoma, which predicts more research heat and value. Early studies have focused on prostate, breast, and ovarian cancers, all of which are positively associated with high cholesterol levels, and the pathogenesis involves the metabolism of steroid hormones such as estrogen, androgens, and progesterone, which are directed to the liver, an LXRα-enriched organ. Antagonistic mechanisms and activation competition between androgens and LXR exist to influence prostate cholesterol levels, and the LXR agonist T0901317 has been shown to effectively inhibit prostate LNCcaP cell proliferation.^[50,51]^ Estrogen homeostasis as an important factor in breast carcinogenesis is controlled by LXR, and multiple LXR ligands or agonist-activated pathways significantly reduce the proliferation of multiple breast cancer cells, a process that involves the regulation of estrogen receptor and estrogen sulfotransferase and exhibits some degree of liver specificity. The cholesterol derivative 27HC stimulates estrogen receptor to knock down the LXR expression to promote breast cancer metastasis, and this pathway also provides an intrinsic link to the mechanism of ovarian cancer progression.^[52,53]^

In the visualization analysis, hepatocellular carcinoma has received much attention as an important class of cancer cell lines that have been studied in recent years, and the liver itself, as a major lipid- and cholesterol-metabolizing organ, has been closely associated with cancer in the known pathways of the LXR mechanism. Notably, with the overall improvement of global health conditions, the proportion of hepatocellular carcinoma caused by etiologies such as hepatitis and aflatoxin has declined, replaced by hepatocellular carcinoma caused by high-fat factors such as nonalcoholic fatty liver disease,^[54]^ Nonalcoholic fatty liver disease is also reflected as an important cluster in the term clustering. Many studies have revealed the crosstalk mechanism between multiple signaling pathways and LXR in hepatocellular carcinoma progression, such as the crosstalk of LXR by TGFβ under the Snail factor contributes to the activation of mesenchymal transition process by hepatocyte apoptosis, which enhances the aggressiveness of hepatocellular carcinoma^[55]^; LXR activates and weakens the phosphorylation process of protein kinase and induces the accumulation of ABCG1 to promote efflux cholesterol and reduce the function of lipid rafts, thereby leading to the inactivation of Akt pathway and interfering with the progression of hepatocellular carcinoma through PI3K/Akt pathway.^[9,38]^ In addition, LXR inactivation of the Wnt/β-catenin pathway largely reduces the progression of hepatocellular carcinoma mesenchymal transition and inhibits tumor metastasis. This process was also found to inhibit the proliferation of gastric cancer cells and promote good differentiation of gastric cancer cells.^[8,56,57]^ The regulation of microRNAs through the LXR pathway has also been shown to be feasible for inhibiting drug resistance in hepatocellular carcinoma-targeted drugs.^[58]^ While developing LXR ligands as possible therapeutic tools based on the anticancer mechanism of LXR has made some progress in several cancer studies, the classical ligand agonist T0901317 possesses a wide range of anticancer mechanisms, targeting agonistic LXR to induce cystathionine-1-dependent apoptosis in colon cancer.^[59]^ LXR ligands activate the LXRβ/ApoE pathway to inhibit melanoma multiple spectrums of growth and proliferation,^[33]^ and the ligand GAC0001E5 targets LXR to produce anti-proliferative effects on pancreatic cancer cells,^[60]^ but of course, these ligand mechanisms need further exploration. In addition to the current studies on several types of cancers, we hope to see a systematic investigation of the mechanism of LXR in more cancer cell lines of different tissues in the future, which will be beneficial to revealing more about cancer.

In addition, at present, the role of LXR in lipid and cholesterol metabolism is relatively clear, but the more exact mechanism of action such as cholesterol homeostasis in cancer progression, as well as whether LXR has other different response pathways or forms cross-talk mechanism with more pathways in cancer progression need to be further explored, which is also expected to become a new direction for future research.

(The items of data collection for this project are partially referenced to the existing literature on the type of bibliometric analysis,^[61–65]^ and are shown in conjunction with the actual discussion needs.)

## 4. Conclusions

In this research, we explored the role played by LXR in cancer through bibliometric analysis to provide an intuitive analysis of the overall status of the topic. In terms of the current state of research: In this study, we explored the role of LXR in cancer through bibliometric analysis to provide a visual analysis of the overall status of the topic. In terms of the current state of research: the research on the mechanisms of LXR and cancer is concentrated in the fields of molecular biology and oncology, and there is a certain degree of interdisciplinary intersections with other disciplines, such as clinical medicine, but it is very limited. Early LXR research in cholesterol metabolism and immunity was the basis for subsequent research, and this early series of basic research was led by institutions in the United States and Europe, while subsequent advances in LXR and multiple cancers or pathways have included a number of institutions from China, and there is also close collaboration between these countries and institutions. Important research results in the 2 directions of cholesterol metabolism and tumor immunity are produced by highly influential and active scholars through forming relatively independent groups. 2014 to 2018 is an important stage in the development of relevant theories, and abundant theoretical elucidations have been made by studying the mechanism of LXR activation in various cancer tissues or pathways and the influence of LXR on various cancer-related immune pathways. In particular, various immune factors in the tumor microenvironment are the key research objects. Among various types of cancer research, breast cancer and hepatocellular cancer seem to be the more important research targets.

Based on the current status of such research and the analysis done in this study, we make some speculations on the future research direction and focus, which may be the direction worth working on in the future: Promote the research on the anti-cancer mechanism of LXR from the micro-direction of molecular biology to the transformation of clinical medicine, and to form a benign circle of evidence-based medical model, the current results of the clinical anti-cancer application of LXR have received some attention, but this is far from being sufficient. Further explore the effects of LXR on the tumor microenvironment as a whole and on more immune pathways. Tumor immunity has been at the forefront in recent years, but the effects of LXR on this seem to be partially controversial, and the results of the studies are relatively independent of each other, which suggests that the role of LXR on the overall orientation of the tumor microenvironment needs to be clarified. A more comprehensive study should be conducted on more cancer cell lines and the crosstalk effect of LXR on cancerous signals. It is known that the LXR activation pathway has a wide applicability to different cancers, but the mechanism of activation is different, we expect that the commonality of the pathways can be found to help explore the new mechanism of cancer. At the same time, the crosstalk between different pathways together constitutes the cancer signal network, which makes the influence of LXR on the cancer pathway urgently need to be more holistic.

## 5. Deficiency and limitation

There are still some limitations in this study, although we made some control on the relevance of the article content to this study in the article selection stage, there is no way to guarantee the quality of each article, the results of keywords and terms are not related to the weights generated by the quality of the article, the existence of some scholars’ self-citation of the literature in the complex citation network may also have some influence on the final results, and the content extracted based on linguistic descriptions may also have some potential deviations, which may need some more reasonable and feasible research methods to deal with. Nevertheless, based on the bibliometric analysis method, we tried to maintain the rationality and accuracy of the study as much as possible, in order to help scholars, researchers, and, medical professionals in related fields to make useful explorations.

## Author contributions

**Conceptualization:** Mingzhang Guo.

**Data curation:** Yukun Chen, Siqi Deng, Jiexia Xu, Mingzhang Guo.

**Investigation:** Yukun Chen, Siqi Deng, Jiexia Xu.

**Methodology:** Mingzhang Guo.

**Software:** Yu Yan, Shuwen Lan.

**Validation:** Yukun Chen.

**Visualization:** Yu Yan, Shuwen Lan.

**Writing – original draft:** Yukun Chen.

**Writing – review & editing:** Mingzhang Guo.

## References

[1] Sung H Ferlay J Siegel RL. Global cancer statistics 2020: GLOBOCAN estimates of incidence and mortality worldwide for 36 cancers in 185 countries. CA Cancer J Clin. 2021;71:209–49. 33538338 10.3322/caac.21660

[2] Bian X Liu R Meng Y. Lipid metabolism and cancer. J Exp Med. 2021;218:e20201606. 33601415 10.1084/jem.20201606 PMC7754673

[3] Cheng C Geng F Cheng X. Lipid metabolism reprogramming and its potential targets in cancer. Cancer Commun (Lond). 2018;38:27. 29784041 10.1186/s40880-018-0301-4 PMC5993136

[4] Bacci M Lorito N Smiriglia A. Fat and furious: lipid metabolism in antitumoral therapy response and resistance. Trends Cancer. 2021;7:198–213. 33281098 10.1016/j.trecan.2020.10.004

[5] Patel MB Oza NA Anand IS. Liver x receptor: a novel therapeutic target. Indian J Pharm Sci. 2008;70:135–44. 20046702 10.4103/0250-474X.41445 PMC2792482

[6] Gabitova L Gorin A Astsaturov I. Molecular pathways: sterols and receptor signaling in cancer. Clin Cancer Res. 2014;20:28–34. 24158702 10.1158/1078-0432.CCR-13-0122 PMC3859141

[7] Ji L Zhang B Zhao G. Liver X receptor α (LXRα) promoted invasion and EMT of gastric cancer cells by regulation of NF-κB activity. Hum Cell. 2017;30:124–32. 28091828 10.1007/s13577-016-0157-3

[8] Wang Q Feng F Wang J. Liver X receptor activation reduces gastric cancer cell proliferation by suppressing Wnt signalling via LXRβ relocalization. J Cell Mol Med. 2019;23:789–97. 30338932 10.1111/jcmm.13974 PMC6349166

[9] Pattanayak SP Bose P Sunita P. Bergapten inhibits liver carcinogenesis by modulating LXR/PI3K/Akt and IDOL/LDLR pathways. Biomed Pharmacother. 2018;108:297–308. 30227322 10.1016/j.biopha.2018.08.145

[10] Joseph SB Bradley MN Castrillo A. LXR-dependent gene expression is important for macrophage survival and the innate immune response. Cell. 2004;119:299–309. 15479645 10.1016/j.cell.2004.09.032

[11] Joseph SB Castrillo A Laffitte BA. Reciprocal regulation of inflammation and lipid metabolism by liver X receptors. Nat Med. 2003;9:213–9. 12524534 10.1038/nm820

[12] Chen C Song M. Visualizing a field of research: a methodology of systematic scientometric reviews. PLoS One. 2019;14:e0223994. 31671124 10.1371/journal.pone.0223994 PMC6822756

[13] Zelcer N Hong C Boyadjian R. LXR regulates cholesterol uptake through Idol-dependent ubiquitination of the LDL receptor. Science. 2009;325:100–4. 19520913 10.1126/science.1168974 PMC2777523

[14] Bensinger SJ Bradley MN Joseph SB. LXR signaling couples sterol metabolism to proliferation in the acquired immune response. Cell. 2008;134:97–111. 18614014 10.1016/j.cell.2008.04.052 PMC2626438

[15] Jakobsson T Treuter E Gustafsson JA. Liver X receptor biology and pharmacology: new pathways, challenges and opportunities. Trends Pharmacol Sci. 2012;33:394–404. 22541735 10.1016/j.tips.2012.03.013

[16] Wu Y Yu DD Yan DL. Liver X receptor as a drug target for the treatment of breast cancer. Anticancer Drugs. 2016;27:373–82. 26872310 10.1097/CAD.0000000000000348

[17] Cao H Yu S Chen D. Liver X receptor agonist T0901317 reverses resistance of A549 human lung cancer cells to EGFR-TKI treatment. FEBS Open Bio. 2016;7:35–43. 10.1002/2211-5463.12147 PMC5221460 28097086

[18] Chen H Chen Z Zhang Z. Discovery of new LXRβ agonists as glioblastoma inhibitors. Eur J Med Chem. 2020;194:112240. 32248003 10.1016/j.ejmech.2020.112240

[19] Shao W Zhu W Lin J. Liver X receptor agonism sensitizes a subset of hepatocellular carcinoma to sorafenib by dual-inhibiting MET and EGFR. Neoplasia. 2020;22:1–9. 31751859 10.1016/j.neo.2019.08.002 PMC6911865

[20] Zhou J Febbraio M Wada T. Hepatic fatty acid transporter Cd36 is a common target of LXR, PXR, and PPARgamma in promoting steatosis. Gastroenterology. 2008;134:556–67. 18242221 10.1053/j.gastro.2007.11.037

[21] Lee JH Zhou J Xie W. PXR and LXR in hepatic steatosis: a new dog and an old dog with new tricks. Mol Pharm. 2008;5:60–6. 18072748 10.1021/mp700121u

[22] Lee JH Gong H Khadem S. Androgen deprivation by activating the liver X receptor. Endocrinology. 2008;149:3778–88. 18450964 10.1210/en.2007-1605 PMC2488233

[23] Walczak R Joseph SB Laffitte BA. Transcription of the vascular endothelial growth factor gene in macrophages is regulated by liver X receptors. J Biol Chem. 2004;279:9905–11. 14699103 10.1074/jbc.M310587200

[24] Villablanca EJ Raccosta L Zhou D. Tumor-mediated liver X receptor-alpha activation inhibits CC chemokine receptor-7 expression on dendritic cells and dampens antitumor responses. Nat Med. 2010;16:98–105. 20037595 10.1038/nm.2074

[25] Vedin LL Lewandowski SA Parini P. The oxysterol receptor LXR inhibits proliferation of human breast cancer cells. Carcinogenesis. 2009;30:575–9. 19168586 10.1093/carcin/bgp029

[26] Ma L Wang L Nelson AT. 27-Hydroxycholesterol acts on myeloid immune cells to induce T cell dysfunction, promoting breast cancer progression. Cancer Lett. 2020;493:266–83. 32861706 10.1016/j.canlet.2020.08.020 PMC7572761

[27] He S Ma L Baek AE. Host CYP27A1 expression is essential for ovarian cancer progression. Endocr Relat Cancer. 2019;26:659–75. 31048561 10.1530/ERC-18-0572 PMC6824983

[28] Sharma B Agnihotri N. Role of cholesterol homeostasis and its efflux pathways in cancer progression. J Steroid Biochem Mol Biol. 2019;191:105377. 31063804 10.1016/j.jsbmb.2019.105377

[29] Lin CY Gustafsson JA. Targeting liver X receptors in cancer therapeutics. Nat Rev Cancer. 2015;15:216–24. 25786697 10.1038/nrc3912

[30] Abdalkareem Jasim S Kzar HH Haider Hamad M. The emerging role of 27-hydroxycholesterol in cancer development and progression: an update. Int Immunopharmacol. 2022;110:109074. 35978522 10.1016/j.intimp.2022.109074

[31] Tavazoie MF Pollack I Tanqueco R. LXR/ApoE activation restricts innate immune suppression in cancer. Cell. 2018;172:825–840.e18. 29336888 10.1016/j.cell.2017.12.026 PMC5846344

[32] Willy PJ Umesono K Ong ES. LXR, a nuclear receptor that defines a distinct retinoid response pathway. Genes Dev. 1995;9:1033–45. 7744246 10.1101/gad.9.9.1033

[33] Peet DJ Turley SD Ma W. Cholesterol and bile acid metabolism are impaired in mice lacking the nuclear oxysterol receptor LXR alpha. Cell. 1998;93:693–704. 9630215 10.1016/s0092-8674(00)81432-4

[34] Venkateswaran A Laffitte BA Joseph SB. Control of cellular cholesterol efflux by the nuclear oxysterol receptor LXR alpha. Proc Natl Acad Sci USA. 2000;97:12097–102. 11035776 10.1073/pnas.200367697 PMC17300

[35] Chuu CP Hiipakka RA Kokontis JM. Inhibition of tumor growth and progression of LNCaP prostate cancer cells in athymic mice by androgen and liver X receptor agonist. Cancer Res. 2006;66:6482–6. 16818617 10.1158/0008-5472.CAN-06-0632

[36] Pommier AJ Alves G Viennois E. Liver X Receptor activation downregulates AKT survival signaling in lipid rafts and induces apoptosis of prostate cancer cells. Oncogene. 2010;29:2712–23. 20190811 10.1038/onc.2010.30

[37] Mok EHK Lee TKW. The pivotal role of the dysregulation of cholesterol homeostasis in cancer: implications for therapeutic targets. Cancers (Basel). 2020;12:1410. 32486083 10.3390/cancers12061410 PMC7352357

[38] Jeon TI Osborne TF. SREBPs: metabolic integrators in physiology and metabolism. Trends Endocrinol Metab. 2012;23:65–72. 22154484 10.1016/j.tem.2011.10.004 PMC3273665

[39] Hong C Marshall SM McDaniel AL. The LXR-Idol axis differentially regulates plasma LDL levels in primates and mice. Cell Metab. 2014;20:910–8. 25440061 10.1016/j.cmet.2014.10.001 PMC4261644

[40] Luo X Cheng C Tan Z. Emerging roles of lipid metabolism in cancer metastasis. Mol Cancer. 2017;16:76. 28399876 10.1186/s12943-017-0646-3 PMC5387196

[41] Gobel A Rauner M Hofbauer LC. Cholesterol and beyond - The role of the mevalonate pathway in cancer biology. Biochim Biophys Acta Rev Cancer. 2020;1873:188351. 32007596 10.1016/j.bbcan.2020.188351

[42] Berndt N Hamilton AD Sebti SM. Targeting protein prenylation for cancer therapy. Nat Rev Cancer. 2011;11:775–91. 22020205 10.1038/nrc3151 PMC4037130

[43] Singh R Mishra MK Aggarwal H. Inflammation, immunity, and cancer. Mediators Inflamm. 2017;2017:6027305. 29234189 10.1155/2017/6027305 PMC5695028

[44] Carbo JM Leon TE Font-Diaz J. Pharmacologic activation of LXR alters the expression profile of tumor-associated macrophages and the abundance of regulatory T cells in the tumor microenvironment. Cancer Res. 2021;81:968–85. 33361391 10.1158/0008-5472.CAN-19-3360

[45] Gonzalez de la Aleja A Herrero C Torres-Torresano M. Activation of LXR nuclear receptors impairs the anti-inflammatory gene and functional profile of M-CSF-dependent human monocyte-derived macrophages. Front Immunol. 2022;13:835478. 35280993 10.3389/fimmu.2022.835478 PMC8907538

[46] Endo-Umeda K Nakashima H Uno S. Liver X receptors regulate natural killer T cell population and antitumor activity in the liver of mice. Sci Rep. 2021;11:22595. 34799646 10.1038/s41598-021-02062-z PMC8604965

[47] Tiwary S Berzofsky JA Terabe M. Altered lipid tumor environment and its potential effects on NKT cell function in tumor immunity. Front Immunol. 2019;10:2187. 31620124 10.3389/fimmu.2019.02187 PMC6759687

[48] Luo Q Zheng N Jiang L. Lipid accumulation in macrophages confers protumorigenic polarization and immunity in gastric cancer. Cancer Sci. 2020;111:4000–11. 32798273 10.1111/cas.14616 PMC7648032

[49] Yan D Adeshakin AO Xu M. Lipid metabolic pathways confer the immunosuppressive function of myeloid-derived suppressor cells in tumor. Front Immunol. 2019;10:1399. 31275326 10.3389/fimmu.2019.01399 PMC6593140

[50] Krycer JR Brown AJ. Cross-talk between the androgen receptor and the liver X receptor: implications for cholesterol homeostasis. J Biol Chem. 2011;286:20637–47. 21489984 10.1074/jbc.M111.227082 PMC3121513

[51] Chuu CP Chen RY Hiipakka RA. The liver X receptor agonist T0901317 acts as androgen receptor antagonist in human prostate cancer cells. Biochem Biophys Res Commun. 2007;357:341–6. 17416342 10.1016/j.bbrc.2007.03.116 PMC2693411

[52] Gong H Guo P Zhai Y. Estrogen deprivation and inhibition of breast cancer growth in vivo through activation of the orphan nuclear receptor liver X receptor. Mol Endocrinol. 2007;21:1781–90. 17536009 10.1210/me.2007-0187

[53] Nazih H Bard JM. Cholesterol, oxysterols and LXRs in breast cancer pathophysiology. Int J Mol Sci. 2020;21:1356. 32079340 10.3390/ijms21041356 PMC7072989

[54] Teng YX Xie S Guo PP. Hepatocellular carcinoma in non-alcoholic fatty liver disease: current progresses and challenges. J Clin Transl Hepatol. 2022;10:955–64. 36304509 10.14218/JCTH.2021.00586 PMC9547250

[55] Bellomo C Caja L Fabregat I. Snail mediates crosstalk between TGFβ and LXRα in hepatocellular carcinoma. Cell Death Differ. 2018;25:885–903. 29230000 10.1038/s41418-017-0021-3 PMC5943406

[56] Matsushita K Morello F Zhang Z. Nuclear hormone receptor LXRα inhibits adipocyte differentiation of mesenchymal stem cells with Wnt/beta-catenin signaling. Lab Invest. 2016;96:230–8. 26595172 10.1038/labinvest.2015.141 PMC4731266

[57] Gao Y Chen Z Wang R. LXRα promotes the differentiation of human gastric cancer cells through inactivation of Wnt/β-catenin signaling. J Cancer. 2019;10:156–67. 30662536 10.7150/jca.28600 PMC6329868

[58] Lin Z Xia S Liang Y. LXR activation potentiates sorafenib sensitivity in HCC by activating microRNA-378a transcription. Theranostics. 2020;10:8834–50. 32754282 10.7150/thno.45158 PMC7392029

[59] Courtaut F Derangere V Chevriaux A. Liver X receptor ligand cytotoxicity in colon cancer cells and not in normal colon epithelial cells depends on LXRβ subcellular localization. Oncotarget. 2015;6:26651–62. 26450852 10.18632/oncotarget.5791 PMC4694942

[60] Srivastava S Widmann S Ho C. Novel liver X receptor ligand GAC0001E5 disrupts glutamine metabolism and induces oxidative stress in pancreatic cancer cells. Int J Mol Sci. 2020;21:9622. 33348693 10.3390/ijms21249622 PMC7767092

[61] Hou Z Wang W Su S. Bibliometric and visualization analysis of biomechanical research on lumbar intervertebral disc. J Pain Res. 2023;16:3441–62. 37869478 10.2147/JPR.S428991 PMC10590139

[62] Li F Zhang D Chen J. Research hotspots and trends of brain-computer interface technology in stroke: a bibliometric study and visualization analysis. Front Neurosci. 2023;17:1243151. 37732305 10.3389/fnins.2023.1243151 PMC10507647

[63] Yang J Wu J Han T. Global research hotspots and frontiers of myasthenia gravis from 2002 to 2021: a bibliometric study. Medicine (Baltimore). 2023;102:e34002. 37327308 10.1097/MD.0000000000034002 PMC10270528

[64] Lu H Han T Li F. Global trends and hotspots in research of robotic surgery in oncology: a bibliometric and visual analysis from 2002 to 2021. Front Oncol. 2022;12:1055118. 36439475 10.3389/fonc.2022.1055118 PMC9691977

[65] Hou Z Jiang P Su S. Hotspots and trends in multiple myeloma bone diseases: a bibliometric visualization analysis. Front Pharmacol. 2022;13:1003228. 36313356 10.3389/fphar.2022.1003228 PMC9614215

